# The Induction of Endothelial Autophagy and Its Role in the Development of Atherosclerosis

**DOI:** 10.3389/fcvm.2022.831847

**Published:** 2022-03-23

**Authors:** Yunqing Hua, Jing Zhang, Qianqian Liu, Jing Su, Yun Zhao, Guobin Zheng, Zhihui Yang, Danping Zhuo, Chuanrui Ma, Guanwei Fan

**Affiliations:** ^1^First Teaching Hospital of Tianjin University of Traditional Chinese Medicine, National Clinical Research Center for Chinese Medicine Acupuncture and Moxibustion, Tianjin, China; ^2^Tianjin State Key Laboratory of Component-Based Chinese Medicine, Tianjin University of Traditional Chinese Medicine, Tianjin, China

**Keywords:** endothelial cells, autophagy, atherosclerosis, oxidative stress, lipophagy

## Abstract

Increasing attention is now being paid to the important role played by autophagic flux in maintaining normal blood vessel walls. Endothelial cell dysfunction initiates the development of atherosclerosis. In the endothelium, a variety of critical triggers ranging from shear stress to circulating blood lipids promote autophagy. Furthermore, emerging evidence links autophagy to a range of important physiological functions such as redox homeostasis, lipid metabolism, and the secretion of vasomodulatory substances that determine the life and death of endothelial cells. Thus, the promotion of autophagy in endothelial cells may have the potential for treating atherosclerosis. This paper reviews the role of endothelial cells in the pathogenesis of atherosclerosis and explores the molecular mechanisms involved in atherosclerosis development.

## Autophagy Permeates the Pathology of Atherosclerosis

### Pathogenesis of Atherosclerosis

Atherosclerosis promotes the development of coronary heart disease (CAD), a major cause of morbidity and mortality throughout the world. The rupture of atherosclerotic plaques and thrombosis are linked to most common cardiovascular diseases, including acute coronary syndrome (ACS), myocardial infarction, and stroke (1). Atherosclerotic pathophysiology involves multiple cell types and related processes, including the activation of endothelial cells (ECs), monocyte recruitment, smooth muscle cell migration, macrophages, and foam cells, as well as instability of the extracellular matrix (ECM). The development of atherosclerosis is linked to various risk factors, including age, sex, and high levels of serum cholesterol; however, uneven and distorted blood flow patterns have been associated with plaque deposition, especially in the branches and bends of the aorta (2). This deposition is related to abnormalities in the structure and function of the endothelium, including damage to the fused elastin layer of the lumen and the uncovering of proteoglycans (3), leading to the accretion of low-density lipoprotein (LDL) which is subsequently oxidized and otherwise modified. Activation of the ECs leads to increased expression of cell adhesion molecules, which bind to circulating pro-inflammatory monocytes. Once monocytes enter the intima, they undergo maturation to macrophages, which express scavenger receptors allowing them to ingest lipoprotein particles, resulting in the formation of “foam cells” (4). These foam cells are known to be an early pathological feature of fatty streaks and atherosclerotic plaques.

### Autophagy in Atherosclerosis

Autophagy is a catabolic process that maintains normal physiological circulation and is intricately involved in the metabolism of nutrients during starvation, the flow of intracellular cargo and mediates cellular life and death. From early to advanced stages of atherosclerotic disease, autophagy profoundly influences the behavior of ECs, macrophages and vascular smooth muscle cells (VSMCs) that determine the course of the lesion (Table 1). In the early stages of the disease, endothelial dysfunction is the initiation point for atherosclerosis. External stimuli (e.g., shear stress, ox-LDL) influence the level and outcome of autophagy in the endothelium, which also responds to the lesion process by regulating oxidative stress, inflammatory responses, death signals and thrombotic factors in reverse. The current studies demonstrate that ox-LDL can activate EC autophagy (17). Upon uptake of ox-LDL, lipids are transported to autophagic vesicles for lysosomal-mediated degradation. At the same time, ox-LDL may also trigger autophagy by inducing endoplasmic reticulum stress (12). Transient knockdown of the essential autophagy gene ATG7 resulted in increased intracellular levels of I-LDL and Ox-LDL, suggesting that in endothelial cells, autophagy may be an important mechanism for regulating excess exogenous lipids (12). It is no coincidence that the magnitude of the effect of shear stress on ECs certainly includes the regulation of autophagy. In response to high shear stress, EC autophagic fluxes are upregulated, which may be mediated by the transcription factors Krüppel like factor (KLF) 2 and KLF4 (18), as well as by Sirt-1 activation of FoxO1 (19). On the other hand, low shear stress induced inhibition of AMPKα and activation of mTORC1, which blocked autophagic flux and eventually observed cell death, senescence, inflammation and favored the development of atherosclerosis. A recent work brings closer the relationship between EC autophagy and atherosclerosis. Natalia Reglero-Real et al. found that ECs inhibit the expression of adhesion molecules on their membranes such as platelet endothelial, cell adhesion molecule-1 (PECAM-1) and VE-cadherin by undergoing intense autophagy in an inflammatory environment (20). And autophagy inhibits tissue infiltration due to transendothelial migration of neutrophils, and the vicious cycle of inflammatory responses that would occur next is stifled. The authors propose that inhibiting monocyte invasion and infiltration into the subendothelium by modulating EC autophagy may push the pause button on the atherogenic process. In conclusion, autophagy may be an effective tool in combating endothelial dysfunction, and the precise regulation of autophagic flux to treat atherosclerosis may also provide new ideas for clinical drug development.

**Table 1:** Genetic studies implicating autophagy in atherosclerosis.

**Genotype**	**Specificity**	**Observations**	**References**
Wip1^−/−^	Whole body	Upregulation of autophagy-dependent cholesterol efflux through the Atm-mTOR-dependent signaling pathway inhibits lipid deposition.	(5)
TFEB^TG^	Macrophage	Reverses autophagic dysfunction of plaques, enhances phagocytosis of p62-rich protein aggregates, inhibits macrophage apoptosis and pro-inflammatory IL-1b levels, thereby reducing atherosclerosis.	(6)
SYK^−/−^	Macrophage	Regulation of MHC-II through enhanced autophagy to reduce IgG levels and inhibit atherosclerosis.	(7)
SR-BI^−/−^	Macrophage	SR-BI deletion reduces autophagy levels by decreasing the base of TFEB, a major regulator of autophagy, and by inhibiting the recruitment of the VPS34-Beclin-1 complex.	(8)
LAMP-2A^−/−^	Macrophage	Blocking the degradation of NLRP3 protein *via* the chaperone-mediated autophagy pathway aggravates the inflammatory response and promotes atherosclerosis	(9)
Arg2^−/−^	Whole body	Knockdown of Arg2 reduces RPS6KB1 levels, enhances PRKAA signaling and aortic endothelial cell autophagy, and reduces atherosclerotic lesion formation.	(10)
Atg14^TG^	Macrophage	Overexpression of ATG14 promoted the fusion of autophagic vesicles with lysosomes, promoted lipid degradation, reduced oxidized Ox-LDL-induced apoptosis and inflammatory responses, and upregulated the number of Treg cells, thereby reducing atherosclerotic lesions.	(11)
	Endothelial cell	Absence of endothelial autophagy significantly increases lipid accumulation within the vessel wall.	(12)
Atg7^−/−^	T cell	Inhibition of T-cell autophagy reduced the number of CD4^+^, CD8^+^ and NKT cells, perhaps reducing atherosclerosis.	(13)
	Vascular smooth muscle cells	Impaired autophagy in SMC promotes the accumulation of debris mitochondria, leading to more oxidative stress and resulting in an unstable plaque phenotype.	(14)
Atg5^−/−^	Endothelial cell	Defective endothelial cell autophagy not only inhibits the alignment of endothelial cells with the direction of blood flow, but also promotes inflammatory, apoptotic and senescent phenotypes.	(15)
	Macrophage	Inhibition of autophagy further activates NLRP3 to promote inflammation and accelerate atherosclerosis	(16)

In the middle stage of atherosclerosis, macrophage autophagy inhibits foam cell formation and inhibits the development of atherosclerosis. It was found that ox-LDL could induce macrophage autophagy through endoplasmic reticulum stress to remove damaged organelles and proteins and to maintain macrophage survival. Autophagy facilitated the degradation process of lipid droplet transport into lysosomes with the efflux of free cholesterol from foam cells and reduced foam cell formation (21). Furthermore, autophagy also influences the polarization of macrophages, and the activation of autophagy promotes the development of macrophages toward the M2 phenotype (22), which exhibits anti-inflammatory properties. As atherosclerosis progresses, cellular autophagy is progressively impaired. In advanced atherosclerosis, autophagy of macrophages is severely impaired, leading to lipid accumulation, impaired mitochondrial clearance and macrophage death with the formation of larger necrotic cores (23). In advanced atherosclerosis, apoptosis of VSMCs, the only cells in the fibrous cap that produce interstitial collagen fibers, inevitably results in reduced collagen fiber synthesis and thinning of the fibrous cap, and therefore largely determines whether the plaque breaks down. Autophagy in VSMCs preserves their activity and protects them from the effects of death. In addition, VSMC phenotype and function are also regulated by autophagy, as evidenced by the secretion of more extracellular matrix and less calcification (24). Taken together, autophagy plays a regulatory role on various cell types during the development of atherosclerosis, and in this review we focus on the dialogue between endothelial cells and their environment through autophagy in the pathological conditions of atherosclerosis.

## EC Activation: The Initiator of Atherosclerosis

**Figure 1:**
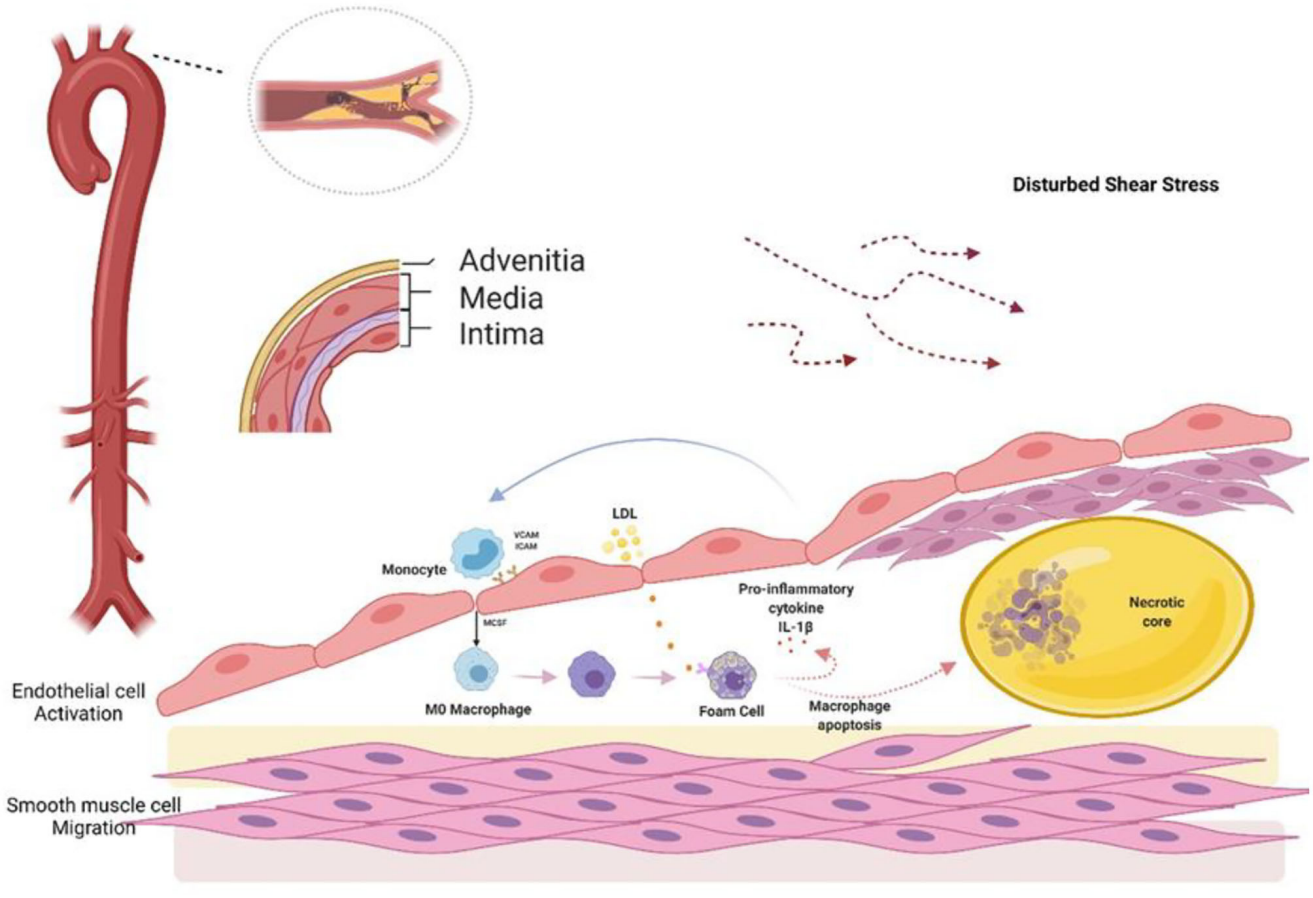
Atherosclerosis begins with endothelial cell activation. The normal arterial wall contains a three-layered structure, with the intima consisting of wellarranged endothelial cells that perform important physiological functions. Endothelial cells normally have anti-inflammatory, anti-proliferative, and antioxidant status. When subjected to shear stress, the endothelium is activated, allowing large numbers of monocytes and LDL to enter the endothelium. In the endothelium, LDL is oxidized, amongst other modifications, and is, in turn, engulfed by macrophages formed by monocyte differentiation to form foam cells. Foam cells secrete pro-inflammatory factors, which promote endothelial cell-expressed ICAM and VCAM-mediated monocyte recruitment, creating a malignant closed loop. Finally, the apoptotic cells and lipids form a necrotic core, accompanied by smooth muscle cells that migrate over the necrotic core to form a fibrous cap. LDL, low-density lipoprotein; ICAM, intercellular cell adhesion molecule-1; VCAM, vascular cell adhesion molecule.

The normal arterial wall has three layers. The outermost layer is the adventitia that contains nerve endings, mast cells, and micro-vessels that nourish the medial membrane. The medial section consists of resting smooth muscle cells and ECM, which includes elastin, collagen, and other macromolecules (25). Atherosclerotic plaques form in the innermost layer—the intima. The intima consists of an EC monolayer arranged in the vascular lumen and is strongly influenced by hemodynamics (25) (Figure 1). ECs are necessary for normal physiological functioning and are involved in many activities, including vasoactive substance processing, neutralization of reactive oxygen species (ROS), lipoprotein transport and breakdown, prostaglandin biosynthesis, and ECM formation and remodeling, as well as interactions with cytokines, growth factors, and hormones (26). In addition, vascular ECs act as both barrier and entry point for the transport of nutrients, gases, and catabolic by-products. They also play a part in the recruitment and accumulation of inflammatory cells, as well as the regulation of vascular tension (27). *In vivo*, ECs are affected by many factors, such as shear stress (28), mechanical stretching (29), lipids, infections (30), and drugs (31) as well as regulating a series of important physiological functions such as permeability, vasodilation, and coagulation.

### ECs Are Regulated by Blood Flow Patterns

The arrangement of ECs along the direction of blood flow was first observed under scanning electron microscopy in 1977 in an atherosclerotic model of hypercholesterolemic rabbits, the first demonstration of the direct effect of physiological laminar flow on the endothelium (32). Subsequently, Frangos et al. investigated the actions of prostacyclin, a vasodilator that blocks both thrombosis and proliferation (33), in primary human EC cultures subjected to a variety of shear stresses, observing that prostacyclin production was greater in cells subjected to disturbed, rather than steady, shear stress (34). The vascular endothelium is extremely susceptible to the effects of shear stress from the circulating blood, which has been found to influence EC mitosis, apoptosis, migration, and nitric oxide release, as well as endodermal permeability, inflammation (including leukocyte adhesion), and thrombosis (35). The endothelial phenotype is strongly influenced by the pattern of blood flow, which explains why atherosclerosis occurs mostly in areas of low shear stress, characterized by vortices and slow back-and-forth oscillations in blood flow (36).

**Figure 2:**
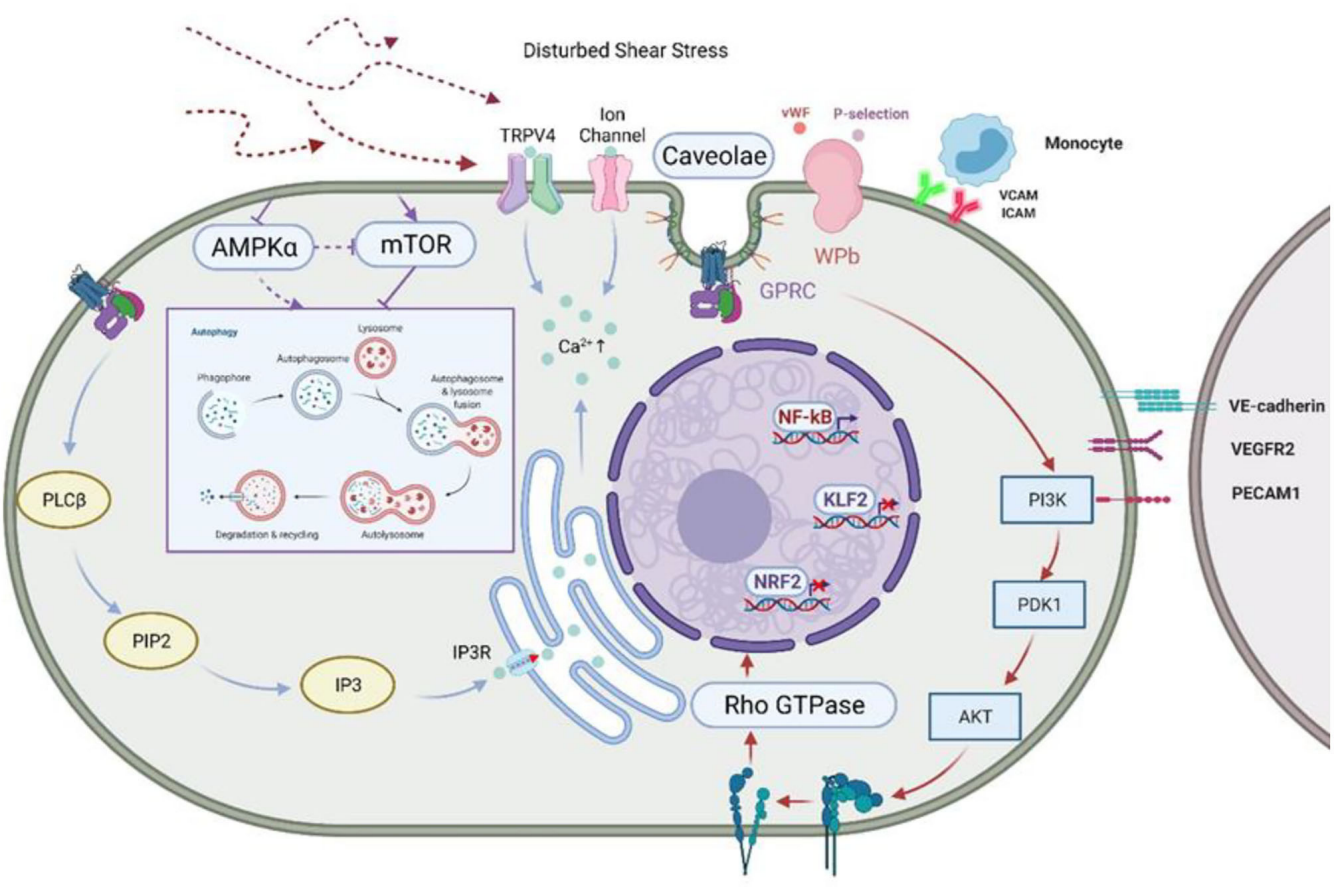
Disturbed shear stress activates ECs and inhibits autophagy. Shear stress inhibits AMPKα and activates mTOR, two classical “autophagy switches,” to inhibit autophagy. Glycocalyxin acts as the first pass-through sensor in the face of shear stress, converting mechanical signals into physiological signals, which are then passed on through ion channels, caveolae, and integrins, amongst others (42). Shear stress regulates endothelial cell behavior through GPCR and the mechano-sensing complex formed by VE-cadherin and VEGFR-2/VEGFR3, which stimulate PI3K/AKT signaling and activate integrins in an “outside-in” manner, leading to the cis-activation of GTPases and the regulation of the expression of various transcription factors (28). This leads to the cis-activation of GTPases and promotes the expression of transcription factors. In addition, stimulated VE-cadherin and VEGFR2/VEGFR3 form a mechano-sensing complex that transmits signals to neighboring cells. At the same time, Ca^2+^ is stored in the endoplasmic reticulum *via* IP3R by the PLCβ/PIP2/IP3 signaling pathway in response to perturbed shear stress. Activation of non-selective cation channels such as TRPV4 and ${\text{K}}_{\text{Ca}}^{2+}$ leads to the influx of more Ca^2+^ and hyperpolarizes the membrane. At the same time, shear stress regulates platelet behavior by inducing the release of P-selectin and vWF from WPBs (46). AMPK, AMP-activated protein kinase; mTOR, mammalian target of rapamycin; GPCR, G protein-coupled receptors; VEGFR, vascular endothelial growth factor receptor; PI3K, phosphatidyl inositol 3 kinase; IP3R, inositol trisphosphate receptor; PLCβ, Phospholipase C Beta 3; PIP2, phosphati-dylinositol-4,5-bisphosphate; IP3, inositol triphosphate; TRPV4, transient receptor potential; ${\text{K}}_{\text{Ca}}^{2+}$, Ca^2+^-sensitive K channels; WPBs, Weibel-Palade bodies; vWF, von Willebrand factor.

*In vitro* studies have demonstrated that shear stress activates a variety of mechanical sensors on the EC membrane. These include integrins (37), tyrosine kinase receptors (especially vascular endothelial growth factor receptor 2) (38), G protein-coupled receptors (39), calcium signals (40) and ion channels (41). Mechanical sensors also include local membrane structures, such as the fossa and gap junction, as well as membrane lipids and the glycolyx (42). The angles between the cells are altered by the flow, resulting in the transmission of tension to adhesion molecules (43) such as platelet and endothelial cell adhesion molecule 1 (PECAM-1) and VE-cadherin and vascular endothelial growth factor receptor 2 (VEGFR2), which activate signal transduction cascades (44). VEGFR2 activation leads to its interaction with vascular endothelial cadherin, β-cadherin-related protein (catenin), and phosphatidylinositol-3 kinase (PI3K), resulting in the downstream phosphorylation of protein kinase B (Akt) (45). This activates integrins which bind to ECM molecules by an inward-out process, activating GTPases and, in turn, transcription factors such as nuclear factor kappa B (NF-κB), Kruppel-like factor 2 (KLF2), and nuclear factor E2-related factor 2 (NRF2/NRF2). This cycle causes further upregulation of VEGFR, influencing both the functioning of the endothelial barrier and the inflammatory response (43) (Figure 2). Other studies have found that the binding of receptor integrin α6β1 to matrix signal protein (CCN1) leads to an accumulation of ROS and the activation of NF-κB, upregulating the expression of a variety of pro-inflammatory and atherogenic genes in ECs. Meanwhile, activation of NF-κB by CCN1/α6β1 results in a positive feedback loop producing more CCN1 and α6β1 (47). There is a great deal of evidence showing the interrelationships between laminar flow and the activation of signaling pathways to regulate EC function.

In particular, the modulation of endothelial cells by laminar flow is also reflected in the regulation of endothelial nitric oxide (eNOS), an important regulator of vascular function. Stable laminar flow exerts atheroprotective effects by promoting NO production, regulating vasotonicity, angiogenesis, anti-inflammation and antithrombosis through upregulation of eNOS (42). Laminar flow regulates the phosphorylation of serines 635, 633, and 1177 on eNOS, together with blocking the Tyr^657^ to regulate nitric oxide (NO) production (48). Phosphorylation of Ser^1177^ is key to eNOS activation and function (49), and can be mediated by AKT (50), Adenosine 5‘-monophosphate-activated protein kinase (AMPK) (51), Ca^2+^/calmodulin dependent protein kinase II (CaMKII) (52), and protein kinase A (PKA) (53). It has recently been reported that shear stress elevates the expression of the scaffold protein MAGI1; this affects EC alignment, promoting the expression of KLF4, eNOS phosphorylation on Ser^1177^, and the release of NO. This MAGI1-induced eNOS phosphorylation is largely performed by PKA and to a lesser extent by AMPK (54). Although steady laminar flow can induce NO production *via* eNOS and increase the level of autophagy in endothelial cells, however, there are now reports demonstrating that NO impairs the level of autophagic flux in a cGMP-independent manner, which are discussed in the following sections (55).

There is now stronger evidence for the control of endothelial cell behavior by shear stress. The regulatory element landscape of endothelial cells was remodeled after only 6 h of stimulation by oscillatory shear stress (OSS). The ETS, Zf and AP1 transcription factor families together with EGR1 and the YAP/TAZ complex are activated by OSS induction. These interactions between TF and OSS-responsive REs lead to intracellular transcriptional changes in the endothelium that regulate cell cycle progression, endothelial cell proliferation and pro-inflammatory responses by promoting inflammation, activating the transforming growth factor-β/Wnt/hippocampal signaling pathway and upregulating YAP/TAZ target genes (56). In turn, all these processes play an integral role in the process of atherosclerotic lesions.

### ECs Activation Caused by Dyslipidemia

One of the signs of atherosclerosis is an increase in plasma ox-LDL levels (57), which leads to endothelial dysfunction (58). This dysfunction is an early indication of atherosclerosis and includes changes in ECs, such as upregulation of adhesion molecules (59), down-regulation of endothelial-dependent vasodilation (60), reduction of NO production (61), and alterations in electrophysiological properties (62).

In 1997, Rangaswamy et al. observed that the endothelial permeability barrier was impaired after intravenous injection of both oxidized and natural LDL into rats (63). Although ECs are in contact with LDL, the mechanisms underlying how the cells react to LDL and how this affects atherosclerosis are not well understood. It has been found that LDL transport across ECs requires an eight-residue cytoplasmic domain of the scavenger receptor class B type 1 (SR-B1) which then recruits the guanine nuclear exchange factor dedicator of cytokinesis 4 (DOCK4) (64). DOCK4 stimulates SR-B1 internalization and LDL transport by complexation of SR-B1 and LDL and activation of the RAS-related C3 toxin substrate1 (RAC1), promoting LDL accumulation by macrophages in the arterial wall, the conversion of macrophages to foam cells, and the development of atherosclerosis (64). Interestingly, both RAC1 and RAC3 also inhibit EC autophagy induced by ox-LDL, increasing the uptake of lipids, together with raising the levels of ROS and stimulating both inflammation and cell membrane dysfunction (65). Lectin-like ox-LDL receptor 1 (LOX-1) is known to mediate ox-LDL uptake by ECs and macrophages. ox-LDL-induced LOX-1 activation triggers endothelial dysfunction and the expression of atherosclerotic inflammatory genes, such as monocyte chemotactic protein 1 (MCP-1), intercellular adhesion molecule 1 (ICAM-1), and vascular cell adhesion molecule 1 (VCAM-1) in ECs (66, 67). Consistent with the above results, LOX-1 overexpression has been found to elevate aortic ox-LDL levels in mice, leading to both endothelial dysfunction and the formation of atherosclerotic plaques. At the same time, this also stimulates p38 phosphorylation, activating NF-κB and, consequently, upregulation of VCAM1, promoting both macrophage and aortic fat streak accumulation (68). Ox-LDL can also stimulate Epithelial sodium channel (ENaC), a negative regulator of vasodilation in ECs, through LOX-1 receptor-mediated NADPH oxidase activation and ROS accumulation (69). Meanwhile, ROS production caused by LOX-1 activation also reduces the utilization rate of NO (70). Furthermore, ox-LDL induces LOX-1-mediated apoptosis of human coronary artery endothelial cells (HCAECs). Jiawei Chen et al. observed that ox-LDL induction of apoptosis involved the downregulation of the apoptosis-inhibitory proteins cellular inhibitor of apoptosis 1 (c-IAP-1) and B-cell lymphoma-2 (Bcl-2), stimulating the release of cytochrome c and sequential model-based algorithm configuration (Smac), activating caspase-9 and caspase-3, and finally inducing EC apoptosis (71).

The Toll-like receptor family members, TLR2 and TLR4, are transmembrane receptors expressed in ECs and are also important targets for ox-LDL-mediated EC inflammation. TLR2 or TLR4 deficiency can prevent insulin resistance and endothelial dysfunction resulting from a high-fat diet (HFD) (72, 73). Upon exposure to ox-LDL, both receptors induce the expression of bone morphogenetic protein 2 (BMP2) through activation of NF-κB and the extracellular regulated protein kinases ERK1/2 (74). Moreover, saturated fatty acids (SFAs) stimulate TLR dimerization, leading to inflammation through the action of myeloid differentiation factor 88 (MyD88) (75). Further studies have shown that the mechanism of palmitic acid and stearic acid-mediated inflammation (to a lesser extent) is not directly linked to TLR4, but involves the conversion to ceramide under the activation of PKC, ERK1/2, JNK (stress-activated protein kinase, SAPK), and p38 (76). These ceramides then sensitize the downstream TLR4 to LPS, amplifying the inflammatory response (76). More interestingly, both ECs and vascular smooth muscle cells can oxidize LDL to some extent during culture, and this “minimally modified” LDL (mmLDL) in turn activates ECs to form a vicious circle by stimulating monocyte adhesion and migration (46). Furthermore, mmLDL induces tyrosine phosphorylation of TLR4, binds to and activates spleen tyrosine kinase (SYK), with downstream activation of the guanosine exchange factors (GEF), and allows the transmission of downstream inflammatory signals (46). In a recent study, Baumer et al. found that ECs ingest and metabolize LDL, but when there is too much cholesterol in the cell, cholesterol crystals (CC) are formed, accumulating on the basolateral side where they impair endothelial function (77). Treatment with the cAMP-enhancer morilyntin/rolipram (F/R) not only improved barrier function but also inhibited the formation of CC *in vitro* and *in vivo*, thereby mitigating the effect of CC on endothelial function (77). However, the mechanism of how increased cAMP expression affects LDL metabolism of ECs still needs to be further explored.

## Autophagy—A Vital Link in the Operation of Cells

In mammals, autophagy forms the main means of degradation, apart from the ubiquitin-proteasome system (78). Autophagy possesses a more powerful degradation capacity, not only targeting pathogens, damaged organelles, aggregated proteins and other structures with larger substances, but also breaking down lipids, DNA, and RNA. In this way, autophagy repels intracellular harmful substances and also provides new pools of amino acids, fatty acids, and nucleosides for anabolic processes and drives a continuous flow of material within the cell in a degradation-regeneration cycle (79). The homeostasis of autophagy is manifested not just in the metabolism of glucose, lipids and amino acids (80), of which there is evidence of a buffering role for oxidative stress and inflammatory bursts (81). These are critical components of metabolic diseases, including obesity, insulin resistance, diabetes and atherosclerosis. There are three main types of autophagy found so far: 1) Chaperone-mediated autophagy, in which heat shock 70 kDa protein 8 (HSPA8/HSC70) recognizes the KFERQ motif, leading to the transport of single misfolded proteins to the lysosomal membrane. 2) Microautophagy, whereby the material to be degraded is directly ingested through the lysosomal membrane. 3) Macroautophagy, the most widely studied form, involving the *de novo* formation of a double-membrane vesicle, the autophagosome, encapsulating and transporting the material to the lysosome (82).

Yet, autophagy may not only cause “autophagic cell death,” which occurs without chromatin condensation and is accompanied by a large number of autophagic vacuoles in the cytoplasm. Notably, autophagy is also inextricably linked to apoptosis and necrosis. Autophagy can send both pro-survival and pro-death signals. The absence of autophagic flux promotes cell death; however, autophagy also provides scaffold for apoptosis and necrosis (83). Autophagy occurs in early atherosclerotic lesions to protect the cells from ROS and inflammation-induced damage. The degradation of faulty proteins and organelles during autophagy normally protects cells from death. During acute or chronic oxidative stress, increased ROS can damage the lysosomal membrane, and oxidative damage to the lysosomal membrane may lead to the release of lysosomal hydrolases, resulting in the degradation of proteins and organelles in the cytoplasm and promoting apoptosis. A large accumulation of ROS can strongly activate autophagy, while over-activated autophagy can also degrade catalase and lead to further accumulation of ROS, creating a vicious cycle. Muller et al. found that sustained or high concentrations of ox-LDL increased the expression of autophagy marker proteins Beclin1 and LC3-II, upregulated cytoplasmic calcium ion concentration, induced endoplasmic reticulum stress, and activated the pro-apoptotic mediators JNK and C/EBP homologous protein (CHOP), resulting in EC apoptosis (84). Apoptotic ECs are pro-coagulant, enhancing platelet adhesion and promoting thrombus formation after plaque rupture. In addition, during severe oxidative stress, autophagy promotes the formation of waxy pigmentation (85). The massive accumulation of waxy pigments in lysosomes disrupts lysosomal hydrolase function and induces the onset of apoptosis. The decrease in lysosomal hydrolase function leads to the accumulation of mitochondria, which further promotes the production of ROS and waxy pigmentation, and exacerbates cell death (86). Thus, the negative effects of autophagy may be related to its over-activation, which in turn may lead to cell death and exacerbate the development of atherosclerosis. A large number of questions remain to be addressed. The question remains whether autophagy-induced survival and death are regulated by the same set of molecular mechanisms; or whether there is a threshold that defines excessive autophagy that exerts detrimental effects. Whether autophagic fluxes differ between early and late atherosclerosis, and how precisely the degree of autophagy is regulated, are crucial.

Autophagy involves the encapsulation of cytoplasmic material in double-membrane vesicles, the autophagosomes, which subsequently fuse with lysosomes to form single-membrane autosomes. The lysosomal hydrolases then degrade the bound material into its constituent parts, such as amino acids, fatty acids, and nucleotides (87) which are then recycled by the cell. Autophagy is controlled by several upstream signaling pathways, the best known of which is that of the nutrient-sensitive Mammalian target of rapamycin (mTOR) kinase. During starvation, reduced cellular energy levels (the ATP/AMP ratio) lead to AMP-dependent phosphorylation of Raptor, which, in turn, leads to leads to TORC1 inhibition (88). The autophagy regulatory pathways characterized to date include the AMPK and SIRTUIN1 pathway, which coordinates the cell energy state and autophagy, regulation of Beclin-1 complex formation with a number of proteins, a link between autophagy and DNA damage provided by p53, and the NF-κB pathway that promotes both inflammation and autophagy (21). Two major autophagic signaling pathways have been identified, namely, one inhibitory, involving class I PI3K-mTOR signaling, and the other inducible, involving class III PI3K-Beclin1 signaling (21). Type I PI3K activates mTOR and mTOR complex 1 (mTORC1) through the Akt pathway when stimulated by growth factors, insulin, or a nutrient-rich environment (89). This, in turn, inhibits autophagy-associated protein 1 (ATG1/ULK1), a protein that initiates the autophagy pathway, and thus prevents autophagosome formation. However, in the presence of nutritional deficiencies and ROS stimulation, complexation between PI3K III and– Beclin-1 is promoted, stimulating the formation of Atg12-Atg5Atg16L complexes and Atg8/LC3 assembly (89). These then promote autophagosome formation and the autophagy process.

The role of autophagy in cardiovascular disease is indispensable. A recent study found that Tax1-binding protein 1 (TAX1BP1) is involved not only in the termination of pro-inflammatory signaling but also in selective autophagy (90). TAX1BP1 recruits ringfinger protein 34 (RNF34), which induces mitochondrial antiviral signaling protein (MAVS) polyubiquitination and autophagic degradation. Autophagic degradation of MAVS inhibits NLRP3 co-localization with mitochondria, thereby avoiding cardiac malfunction due to mitochondrial damage (90). In conclusion, the scope of autophagy regulation goes far beyond our current field of study, making targeting autophagy an effective strategy for the treatment of chronic metabolic diseases such as cardiovascular disease.

## Autophagy—A Bridge Between EC Function and the Occurrence and Development of Atherosclerosis

### Autophagy and Redox Homeostasis in ECs

The perception of hemodynamic shear stress by mechanical sensors in ECs trigger several signaling pathways to ultimately modulate gene and protein expression. Finally, anti-atherosclerosis or pro-atherosclerotic reactions are determined by the effects of blood flow patterns on the vascular wall. The final response depends to a large extent on the redox signals regulated by the interaction between ROS and NO in physiological or pathological processes.

#### Crosstalk Between Autophagy and ROS

**Figure 3:**
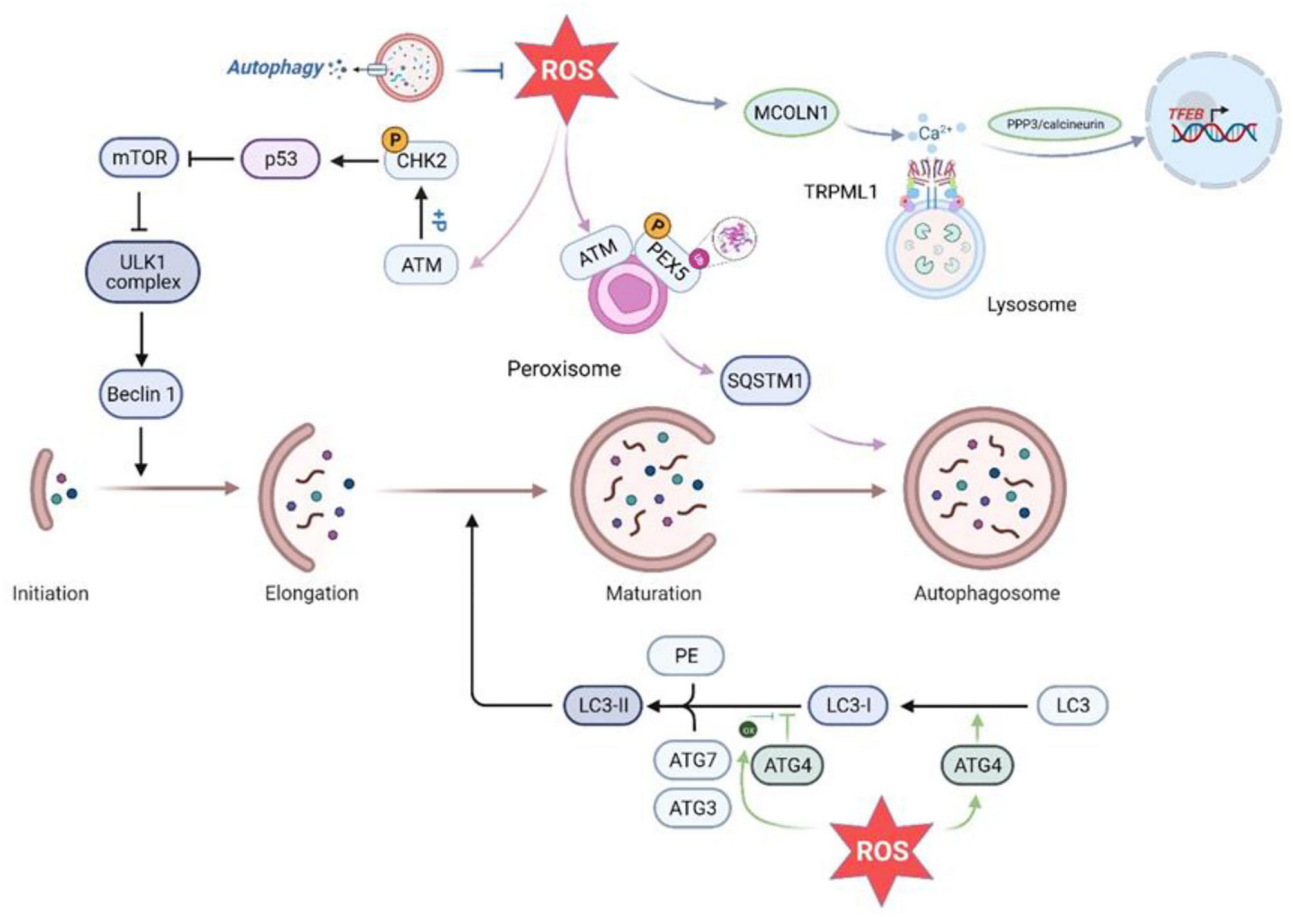
Effects of ROS on various aspects of autophagy. ROS promote autophagy initiation by activating ATM to phosphorylate Chk2 at Ser90/Ser93, which in turn inhibits mTOR. In addition, in the peroxisome, PEX5 is phosphorylated and ubiquitinated in an ATM-dependent manner activated by ROS, promoting autophagy by enhancing SQSTM recognition of the cargo (95). In addition, increased ROS inhibit ATG4-mediated LC3 delipidation and promote autophagic vesicle maturation. Higher levels of ROS lead to ATG4 oxidation and inhibit ATG4 deconjugation of LC3 to allow phagosome expansion and promote autophagic membrane formation. Increased intracellular ROS levels can directly activate Ca^2+^ channels in the lysosomal membrane (MCOLN1/TRPML1), inducing Ca^2+^ release and triggering PPP3/calcineurin-dependent TFEB nuclear translocation to promote autophagy. ROS, reactive oxygen species; ATM, ataxia-telangiectasia mutant; CHK2, cell cycle checkpoint kinase 2; PEX5, peroxisome import receptor; SQSTM1, sequestosome1; ATG, autophagy-related gene; PE, phosphatidylethanolamine; MCOLN1, mucolipin 1;TRPML1, Transient Receptor Potential Cation Channel Subfamily;PPP3, protein phosphatase 3;TFEB, transcription factor EB.

Oxidative stress arises as a result of both pathophysiological conditions and aging. The cell's survival depends on whether the production of oxidants, such as ROS, are able to overwhelm the cell's intrinsic antioxidant and detoxification mechanisms. If the stress is chronic, permanent damage to proteins and cellular structures may result, thus transmitting a “death” signal. However, a moderate elevation of ROS levels activates the cell's “survival” mechanisms, including autophagy, to remove the damaged and oxidized material as well as the excess ROS (91). Where organelles are destroyed by ROS, autophagy and mitosis are activated to degrade and recycle the damaged material, leading to the survival of the cell and the restoration of homeostasis *in vivo* (92). If the organelles are only partially or incompletely degraded, further oxidative stress may result and the ROS-induced ROS release cycle accelerates (92). Almost a decade ago, the study of the effects of hunger, a classic cause of autophagic flux, associated redox biology with autophagy. The response of cells to hunger is complex, involving the activation and inhibition of various signaling pathways. In this case, due to the lack of external nutrition, the cells will carry out autophagy to provide intermediates for biosynthesis (93). Another classic reaction is autophagy triggered by elevated levels of mitochondrial-derived ROS. Cells lacking essential autophagy genes increase their basal ROS levels, thus ROS production occurs upstream of autophagy. The autophagy-associated protein Atg4 is also a molecular targets of hunger-induced ROS (94). Starvation-induced ROS oxidizes key cysteine residues near the Atg4 active site, inhibiting the enzyme and thus transforming Atg8 into a state conducive to autophagosome formation (93) (Figure 3). Moreover, under conditions of starvation, hypoxia, or exercise, the body upregulates the complexation of the mTORC1 inhibitor REDD1 and the pro-oxidant protein TXNIP, inducing ROS production and inhibiting the Atg4-mediated LC3 skimming, and promoting autophagosome maturation (96). This suggests that ROS can act as intracellular messengers to regulate the formation of autophagosomes in autophagy induced by various factors.

Indeed, there are many ways in which ROS induces autophagy, apart from the regulation of Atg4. Transcription factor EB (TFEB) is a major regulator of the lysosomal part of the autophagy pathway and is negatively regulated by mTOR and plays an important role in the induction of autophagy by starvation. After mTOR inhibition, TFEB dephosphorylation causes TFEB to migrate from the cytoplasm to the nucleus, thereby activating the protein. In addition, both endogenous and exogenous ROS levels directly activate MCOLN1/TRPML1, the principal channel for releasing Ca^2+^ in the lysosomal membrane. This induces the release of lysosomal Ca^2+^ and triggers PPP3/calcineurin-dependent translocation of TFEB to the nucleus, promoting autophagy (91) (Figure 3). Thus, ROS may generate autophagy through activation of the MCOLN1-lysosomal Ca^2+^-TFEB pathway, allowing the clearance of both damaged mitochondria and excess ROS. In addition, in another study, the ataxia-telangiectasia mutant (ATM) kinase in peroxisomes can be activated by peroxisomal ROS, resulting in phosphorylation of the peroxisome import receptor (PEX) (93). This ATM-induced phosphorylation leads to the ubiquitination of PEX5, which is recognized by the autophagy ligator p62 (93). Therefore, there is a possible steady-state cycle in which the increase in peroxisomal ROS triggers the activation of ATM, resulting in PEX5 phosphorylation and ubiquitination, and finally activating pexophagy, a subclass of selective autophagy. Recent studies have also demonstrated significant effects of ROS on autophagy, showing that it regulates autophagy through ATM and cell cycle checkpoint kinase 2 (CHK2). CHK2 binds and phosphorylates Beclin-1 at Ser90/Ser93, thereby preventing the formation of the autophagy inhibitory complex Beclin-1-Bcl-2 in a ROS-dependent manner (97). CHK2-induced autophagy ROS-ATM-CHK2-Beclin1 autophagy axis restricts the intracellular ROS level by clearing damaged mitochondria, and protects cells exposed to pathological conditions from stress-induced tissue damage (97).

On the other hand, Mitochondria are a source of ROS and a major organelle for ROS attack. Excess ROS in the cytosol also initiates mitochondrial autophagy to avoid further damage. Mitochondria are the center of cellular energy metabolism, where oxidative phosphorylation, fatty acid oxidative production and other energy metabolism occur, as well as the regulatory center of apoptosis. Progressive reduction in respiratory chain enzyme activity, excessive ROS production and accumulated mtDNA damage or mutations in response to mitochondrial dysfunction are all closely associated with the development of atherosclerosis. Excessive ROS-mediated oxidative stress promotes EC senescence (98) and increases the permeability of the vascular EC membrane (99), thereby compromising the barrier function of the endothelium. Moreover, transient opening of mPTP depolarizes the mitochondrial membrane potential, while prolonged opening of mPTP leads to matrix swelling, rupture of the outer mitochondrial membrane, ATP hydrolysis, and induction of apoptosis, which facilitates plaque rupture (100). Mitophagy is an autophagic process that selectively removes excess or damaged mitochondria and plays an important role in regulating intracellular mitochondrial number and maintaining normal mitochondrial function. It has been found that ox-LDL mediates EC apoptosis by inhibiting mitophagy (101). Interestingly, mitophagy contributes to mediating endothelial cell adaptation to gravitational unloading by maintaining metabolic homeostasis (102). This is reminiscent of the existence of a mechanism associated with mitophagy in ECs under shear stress to maintain cell survival, although this has not yet been reported. Recent studies have revealed that inhibition of phosphatase and tensin homolog deleted on chromosome 10 (PTEN) can reduce EC apoptosis and protect endothelial function in patients with coronary artery disease by regulating the AMPK-CREB-MFN2- mitophagy signaling pathway (103). The role of mitophagy in vascular endothelial injury and atherosclerosis and its mechanisms are still not fully understood and are mostly at the laboratory stage, while clinical studies and evidence for disease treatment are insufficient. The results of these studies provide a theoretical basis for their relevance and still need to be further explored.

#### Endothelial Autophagy and NO Synthesis

The endothelium releases NO in response to shear stress, and NO modulates the vascular environment by inhibiting inflammatory factors, such as cytokines secreted by ECs in the vascular wall and associated platelets, as well as growth factors and cell adhesion molecules. Autophagy is responsible not only for maintaining the levels of NO but also for regulating the redox balance and the balance between pro- and anti-inflammatory factors, all of which contribute to the EC response to shear stress (104). It is welldocumented that ROS functions upstream of autophagy and, indeed, regulates autophagy. However, the precise parts played by reactive nitrogen species, such as NO, in autophagy are not clear. Bharath et al. found that ECs in which Atg3 had been silenced showed severely dysfunctional nitric oxide synthase phosphorylation and could not increase NO production in response to shear stress. In addition, inhibition of autophagy also leads to the accumulation of ROS and the increase of inflammatory cytokines, such as monocyte chemoattractant protein-1(MCP-1) and interleukin-8 (IL-8) (104). *In vitro* perfusion of vascular endothelial cells, stable laminar shear stress increased both autophagy and the levels of eNOS, while inhibiting the expression of endothelin-1 (ET-1). Moreover, compared with the stable lamellar stress treatment alone, autophagy induction by rapamycin pretreatment further up-regulated eNOS and decreased ET-1 in endothelial cells. These results indicate that under stable laminar shear stress, autophagy is increased and the maintenance of vascular tension by ECs is improved (105). Under the induction of LSS, the levels of the autophagy markers Beclin-1 and LC3II/LC3I in ECs were significantly decreased, while that of the autophagy substrate p62 was increased. Meanwhile, the expression of Ten-eleventranslocation2 (Tet2), which promotes autophagy in ECs, was also significantly down-regulated. In addition, TET2 overexpression up-regulated eNOS expression while downregulating that of ET-1 (106). In conclusion, Tet2, down-regulated by LSS, may be a critical target for regulating EC autophagy and eNOS expression. Importantly, inhibition of autophagy in healthy ECs impairs the activation of eNOS to a similar extent to that seen in ECs from diabetic patients. In the latter, spermidine-activated autophagy restored insulin-mediated eNOS activation and enhanced NO production in ECs cultured under high-glucose conditions (107). Impairment of endothelial autophagic flux in response to LSS stimulation led to eNOS Thr495 phosphorylation and eNOS uncoupling, which inhibited NO production and caused massive ${\text{O}}_{2}^{.-}$accumulation (108).

Autophagy plays a role in promoting the production of NO through eNOS. However, it has been found that NO inhibits autophagy by two different mechanisms independent of cGMP. First, NO inactivates JNK1, thereby reducing Bcl-2 phosphorylation and inhibiting Beclin 1-hVps34 binding by increasing Bcl-2-Beclin 1 interactions. Secondly, activation of mTORC1 by NO in a TSC2 and IKKB-dependent manner also exerts autophagy inhibition (55). Hence the complex relationship between autophagy and NOS remains to be investigated. Whether the laminar flow-induced increase in NO levels is a direct result of increased autophagy through increased levels of autophagy or whether there are other cellular processes and signaling cascades involved. Not only can NOS negatively regulate autophagy through NO production by affecting the JNK1-Bcl-2-Beclin1 and IKK-AMPK-TSC2-mTOR pathways, but NOS itself can also directly impair autophagosome formation *via* the JNK1-Bcl-2 pathway (55). This suggests that there may be additional, unidentified pathways by which NO regulates autophagy. Another possibility is that NOS directly affects autophagic mechanisms downstream of these signaling pathways. Since eNOS has a significant impact on endothelial cell behavior, the authors suggest that the crosstalk between autophagy and NOS deserves further investigation, and that it is worthwhile to consider whether the inhibitory effect of NO and NOS on autophagy is a protective effect against excessive autophagy through negative feedback.

### Autophagy and Lipophagy

**Figure 4:**
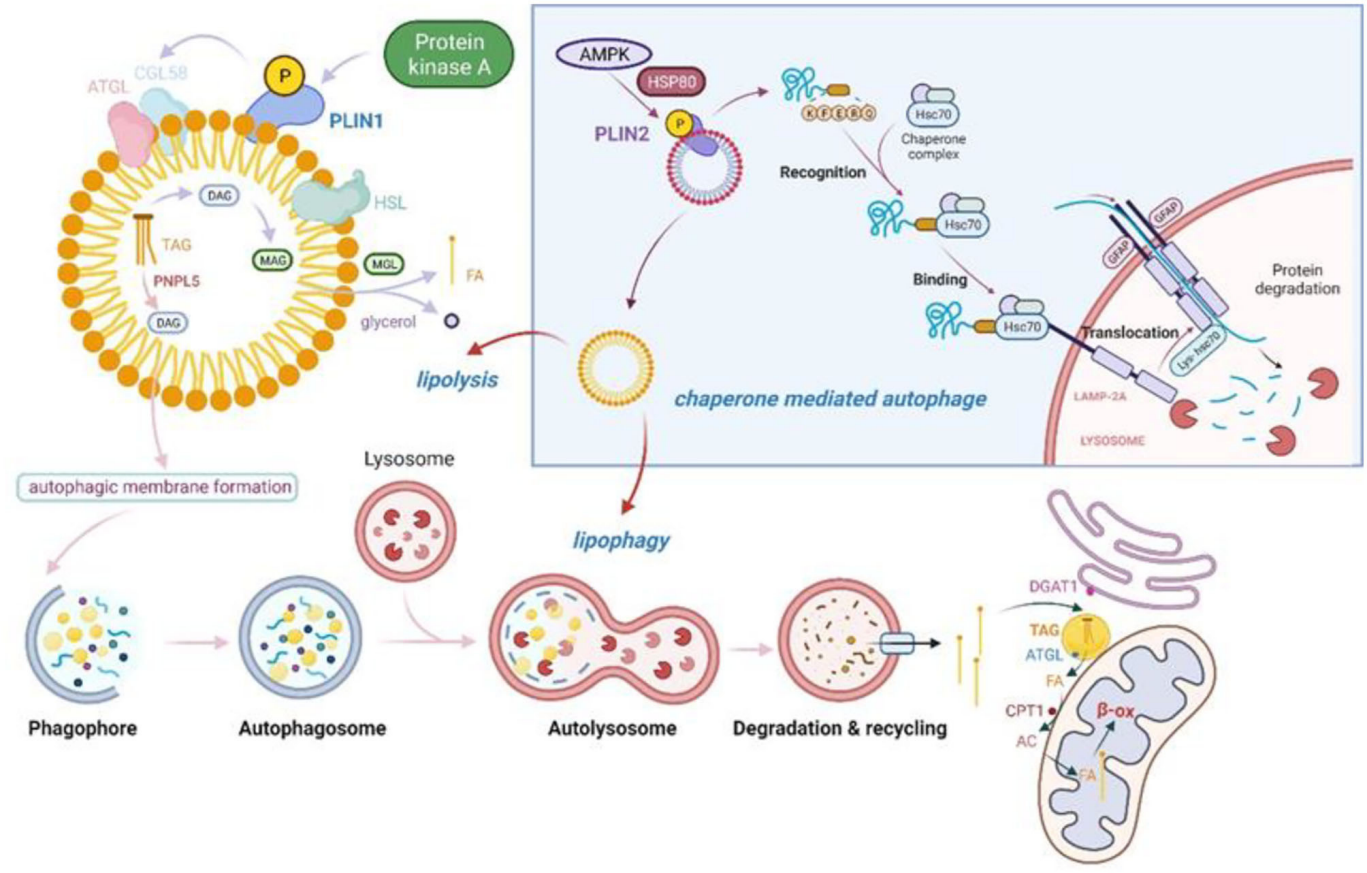
Lipolysis and lipophagy in tandem with molecular chaperone-mediated autophagy (CMA). PKA phosphorylates PLIN1, a member of the PAT protein family, and causes its degradation. This induces the release of CGI-58, which then initiates TAG catabolism *via* activated ATGL. ATGL catalyzes the first step of TAG hydrolysis, producing DAG and free fatty acids. The second step of lipolysis is dependent on the activation of HSL, the rate-limiting enzyme in the lipolysis cascade reaction (113). HSL hydrolyses DAG to produce monoacylglycerols and FFAs, which are excreted from LDs and enter the mitochondria for β-oxidation (113). In addition, the novel neutral lipase PNPLA5 in LDs has triglyceride hydrolase activity and can hydrolyze TAG to DAG, promoting autophagic vesicle membrane formation in a PNPLA5-dependent manner. Furthermore, LDs are also present as a 'staging area' for the entry of FFAs into mitochondria for β-oxidation, thus protecting them from damage by toxic ACs and excess FFAs (114). CMA may be an important link in the initiation of lipophagy and lipolysis. AMPK phosphorylates PLIN2 on LDs in an HSP80-dependent manner, allowing it to be degraded by lysosomes by way of CMA. The degradation of PLIN2 allows lipid droplets to undergo subsequent lipophagy and lipolysis. PKA, protein kinase A; PLIN, perilipin; CGI-58, comparative gene identification-58; TAG, triacylglycerides; ATGL, adipose triglyceride lipase; DAG, diacylglycerol; HSL, hormone-sensitive lipase; FFAs, free fatty acids; LDs, lipid droplets; PNPLA5, Patatin-like phospholipase domain containing 5; DGAT1, diacylglycerol acyltransferase 1; CPT1, carnitine palmitoyltransferase 1; AC, acylcarnitine.

Lipids are not only important organic components but are also principally responsible for energy storage. They are usually found in the cytoplasm as lipid droplets (LDs) with diameters between 0.1 and 100 μm. The LD core consists primarily of neutral lipids (mainly triacylglycerol [TG] and sterol esters), while the outer layer is formed by a phospholipid monolayer and numerous lipoproteins, mostly belonging to the periplasmic lipoprotein family PLIN (109). Under starvation conditions, cells are supplied with energy through the breakdown of LDs into free fatty acids (FFAs), which are then translocated to the mitochondria, resulting in a shift in cellular metabolism from glycolysis to fatty acid oxidation. However, excess FFAs in the cytoplasm can cause lipotoxicity through ROS production, which, in turn, can cause damage to mitochondria and other cellular components (110). The excess intracellular FFAs are then transformed back into LDs, in order to maintain intracellular lipid homeostasis (110). Singh et al. found that large amounts of LDs accumulated in cultured hepatocytes after treatment with 3-methyladenine (3-MA) or the knockdown of Atg7 and Atg5 by shRNA, accompanied by a significant reduction in fatty acid oxidation (111). Moreover, the hepatocyte LDs underwent autophagy under conditions of lipid accumulation (111). This is the first time that LDs were found to be degraded by autophagy, and this phenomenon was named lipophagy. Numerous subsequent studies have confirmed that cells can use two main LDs catabolic pathways: lipolysis *via* cytosolic neutral lipases such as PNPLA2/ATGL (containing the structural domains of two paratetin-like phospholipases) and the autophagic/lipophagic pathway (112) (Figure 4).

Figure 4 shows that in starving cells, FAs are not transported directly to the mitochondria for energy production through β-oxidation after being released through lipophagy but are instead returned to the endoplasmic reticulum. In the endoplasmic reticulum, the FAs are synthesized into triacylglycerols (TAGs) by diacylglycerol acyltransferase 1 (DGAT1), which are stored in endoplasmic reticulum-derived LDs (115). In addition, the TAGs stored in the LDs are used by the lipolytic enzyme aliphatic triglyceride lipase (ATGL) to generate FAs, which are then converted to acylcarnitine (AC) under the action of carnitine palmitoyl transferase 1 (CPT1) and transported to the mitochondria, where they generate ATP by β-oxidation (114). At this point, the LDs act as “staging post stabilizers,” effectively avoiding the mitochondrial threat caused by excess FAs in the cytoplasm. The ability of LDs to buffer fatty acids becomes a key determinant of autophagy regulation and cellular stress resistance.

However, the mechanism of lipophagy, and, in particular, how autophagy selectively recognizes LDs for degradation, remains unknown. It has been shown that LDs enhance autophagy in mammalian cells. One of the uses of lipids stored in LDs is membrane synthesis, and this appears to apply also to autophagic membrane synthesis. The patatin-like phospholipase domain containing 5 (PNPLA5) enzyme is a novel neutral lipase that is localized to LDs and has triglyceride hydrolase activity (116). Diacylglycerol (DAG), a potential intermediate formed by PNPLA5 through the action of TGs mobilized from LDs, can be used for membrane formation in autophagic vesicles (117). The Shpilka et al. found that deletion of the Dga1 and Lro1 enzymes responsible for the synthesis of triacylglycerol (TAG) or the Are1 and Are2 enzymes responsible for the synthesis of sterol esters (STE) prevented autophagy. It is also possible that the endoplasmic reticulum-binding proteins Ice2 and Ldb16 may regulate autophagy in an LD-dependent manner, firther evidence for the role of LDs in stimulating autophagy (118).

The process of LD degradation is not straightforward, and the LD surface is associated with hundreds of proteins that are implicated in both the metabolic modulation and lipid signaling activities of LDs. The most important are the five-member PLIN family proteins, PLIN1-5. PLIN2 and PLIN3 both contain the KFERQ chaperone-mediated autophagy recognition motif, which binds to the 70 kDa heat shock-associated protein (Hsc70) and directs LDs toward the lysosome, *via* another type of autophagy —chaperone-mediated autophagy (CMA)—for degradation (119).

Although CMA is responsible for protein, rather than lipid, degradation, it is required for LD lipolysis and lipophagy. It has been shown that CMA breaks down the LD proteins exolipin PLIN2 and 3 on the LD surface, recruiting both cytosolic lipase and autophagic effectors to the LDs. PLIN2 phosphorylation occurred at the initiation of lipolysis, and was shown to be AMPK-dependent (120). HSPA8 bound to PLIN2 on the LD surface, leading to its phosphorylation (120) after which it was released and degraded by CMA (120). This led to the recruitment of the PNPLA2/ATGL and ATG protein, resulting in the breakdown of the LD triglycerides by PNPLA2 or the delivery of part of the LD to the lysosome for degradation by macroautophagy, mediated by ATG proteins (120).

It remains to be explored whether lipophagy is responsible for degrading excess intracellular lipids in ECs, which would limit their availability to macrophages, although it is known that ox-LDL stimulates the formation of autophagosomes. Furthermore, confocal and electron microscopy have shown the apparent engulfment of both natural and modified LDL in autophagosomes, and greater concentrations of intracellular ^125^I-LDL and ox-LDL were observed after knockdown of the autophagy-promoting ATG7 gene (12). Besides, when crossed with mice with endothelial-specific knockout of the ATG7 gene compared to ApoE^−/−^ mice, it resulted in more lipid deposition with larger plaque area in the aorta (12). This suggests that autophagy may play a significant role in the regulation of excess lipid in ECs.

### Autophagy Regulates the Exocytosis of ECs

ECs are unique in containing Weibel-Palade bodies (WPBs), long secretory organelles that contain von Willebrand Factor (vWF) and regulatory factors such as P-selectin, interleukin-8, angiopoietin 2, and endothelin 1 (121). Leukocytes secrete vWF after damage to blood vessels, which stimulates hemostasis and binds to circulating platelets to form sparse blockages. vWF is also known to be a risk factor for arterial thrombosis (122). It stimulates both leukocyte-EC interactions and leukocyte infiltration into inflammatory tissue, both of which promote atherosclerosis development (123). Furthermore, vWF deficiency protects against atherosclerosis in arterial branches (124). These findings suggest that vascular vWF may be a potential target for the prevention of atherothrombosis and atherosclerosis.

Ox-LDL has been shown to increase the secretion of vWF and P-selectin in HUVEC, which is associated with the role of ox-LDL in inhibiting the Sirt1/FoxO1 pathway and autophagic flux in HUVEC, suggesting that increased autophagic flux may be a potential target for reducing inflammation and thrombotic risk (122). However, contrary findings have also been reported, where pharmacological autophagic inhibitors or silencing of the ATG5 and ATG7 genes blocked vWF secretion *in vitro* (125). Furthermore, although endothelial-specific ATG7-deficient mice have normal vascular and capillary structures, the mice were found to display reduced vWF secretion and levels of high molecular weight vWF oligomers, resulting in increased bleeding times (125). There is clinical evidence that the use of rapamycin-eluting stents elevates the risk of stent thrombosis by stimulation of tissue factor (TF), leading to increased coagulation, compared to bare metal stents (126). Also, the induction of fibrinogen activator inhibitor 1 (PAI-1) in HUVECs by rapamycin may be critical in causing thrombosis (127). The above phenomenon may be related to other pharmacological effects of rapamycin and does not suggest that this is directly caused by autophagy. However, Pingjiang et al. found that rapamycin-induced endothelial membrane remodeling enhanced the adhesion between platelets and ECs, promoting thrombosis, and that the endothelial membrane remodeling was autophagy-dependent (127). It is unclear whether autophagy is related to cytosolic emesis of Webel-Palade vesicles and the secretion of vWF. The authors suggest that this is limited by the use of agonists and inhibitors of autophagy and the knowledge that knockdown of autophagy-related genes interferes with pathways other than autophagy. Alternatively, one-sided results in different stimuli and *in vitro* and *in vivo* environments have led to opposite findings, and the mechanisms involved need to be further investigated.

Other studies now suggest that autophagy promotes the production and secretion of inflammatory factors, a behavior that extends the function of autophagy beyond self-digestion and quality control in mammals. In mammals, autophagy promotes the synthesis and secretion of the pro-inflammatory factor IL-1β. During autophagy, the inflammasome and autophagic apparatus act synergistically in the secretion of IL-1β through GRASP and Rab8a, a GTPase that controls post-Golgi polarization sorting and cytosolic spitting. This allows cytoplasmic proteins that lack signal peptides and thus cannot be secreted conventionally through the endoplasmic reticulum to be secreted through the autophagy pathway, contributing to increased IL-1β secretion (128). It has been found that cytokine-induced autophagy contributes to the degradation of nuclear factor IκBα, promotes nuclear translocation of NF-κB and the transcription of VCAM-1, raising the levels of VCAM-1 in the long-term and stimulating lymphocyte recruitment and adhesion to the endothelium (117). These findings indicate that autophagy may play important physiological roles other than self-digestion, and that, while the anti-inflammatory role of autophagy is well known (129), the secretory role of autophagy is of significant interest.

## Pharmacological Modulation of Autophagy

To date, there have been a number of drugs approved by the FDA for clinical use in cancer treatment (Rapamycin derivatives, Nilotinib, Bortezomib) through modulation of autophagy and a proportion of autophagy agonists are also in clinical trials for neurodegenerative diseases (130). A large number of clinical trials are associated with everolimus-eluting stents (NCT02389946, NCT00911976, NCT00783796, NCT02681016), whose use results in better hematological reconstitution and reduced inflammation and foreign body reactions. Although clinical trials related to the oral administration of autophagy modulators for the treatment of atherosclerosis are not currently widely available, the results of a large number of basic studies have provided enough evidence for this that we believe it will one day be on the agenda. Trehalose has been shown to promote lysosomal autophagy by activating TFEB nuclear translocation (6), and in another clinical study, oral administration of trehalose reversed arterial aging in middle-aged and elderly people (NCT01575288). Several compounds have been shown to exert vasoprotective effects *via* autophagy in animal models. The antidepressant indatraline inhibits SMC proliferation in restenosis models by targeting the AMPK-TOR-S6 K signaling axis to activate autophagy (131). Sirolimus maintains the integrity of the endothelial barrier in ischemia-reperfusion injury by targeting mTOR to induce autophagy and regulating the localization of the tight junction protein Cldn5 (132). Ebselen acts as a mimic of peroxiredoxin 1 (PRDX1) to maintain lipophagic flux levels and promote cholesterol efflux by reducing ROS production (133). These also remind us whether manipulation of lipophagy in metabolic diseases is also an effective means of treatment. A recent study found that the use of autophagy tethering compounds (ATTECs) not only degrades specific disease-associated proteins through autophagy, but also has a beneficial effect on the clearance of LDs. These compounds were designed by connecting LC3-binding molecules to LD-binding probes *via* a linker and achieved effective clearance of LDs (134). Theoretically all autophagic substrates can be degraded by the designed ATTEC, which provides a new idea for the specific clinical clearance of proteins and lipids by autophagy. The limited application of orally administered mTOR inhibitors to enhance autophagy levels is attributed to the complexity of the signaling pathways regulated by mTOR. Several mechanisms have been proposed for mTOR inhibition-mediated dyslipidemia, including reduced lipoprotein catabolism containing apoB100 (135), impaired bile acid synthesis (136), and inhibition of lipoprotein lipase activity following upregulation of apolipoprotein CIII (137). Statins not only lower lipids but also induce autophagy by activating AMPK and/or inhibiting Rac1 mTOR signaling (138). Linking the two may therefore help to enhance mTOR inhibitor-induced autophagy and maintain normal lipid levels and improve plaque stability. Overall, there is a need to identify not only the precise targets in autophagy and the feasible application of these targets to identify appropriate drugs, but also to monitor autophagic fluxes to ensure drug specificity and safety. In light of the fact that sirolimus (an mTOR inhibitor), nilotinib (an AMPK agonist) and Bortezomib (acting on the 38-MAPK-JUNK pathway) have been approved by the FDA for the treatment of cancer and have achieved significant clinical success, we believe that new drugs for the treatment of cardiovascular disease through the regulation of autophagy will emerge and enter clinical use for the benefit of human health.

## Conclusion

Endothelial cells are the first victims of atherosclerosis, as disturbed shear stress and excess lipids in the blood overwhelm the endothelium and activate it, initiating the pathological process of atherosclerosis. ROS activate autophagy, which in turn maintains the survival of ECs by scavenging excess ROS and degrading misfolded proteins and damaged organelles, as well as maintaining NO bioavailability and regulating vasodilation. CMA also regulates lipophagy and lipolysis, potentially manipulating lipid metabolism as an essential link in the promotion of these processes, and it is worth exploring whether these influence the process by which ECs allow the entry of lipoproteins into the endothelium and their presentation to macrophages. Furthermore, autophagic vesicles are always observed in the vicinity of WPBs, and there is already evidence that autophagy modulates vWF release from WBP vesicles, which inhibits thrombosis. However, it is worth noting that, in contrast to autophagy's accepted role in cell protection, excessive autophagy may be detrimental, leading to adverse consequences and apoptosis. Moreover, some inflammatory factors may be synthesized and secreted in an autophagy-dependent manner, although these mechanisms are unclear. In summary, autophagy broadly regulates the body's secretory, metabolic, and stress processes and determines cellular life and death. Exploring the mechanisms of endothelial autophagy and finding appropriate pro-autophagic drugs may provide new therapeutic targets for atherosclerosis.

## Author Contributions

YH wrote this manuscript, which was suggested by JZ and revised by GF and CM. All authors reviewed and edited this manuscript. All authors contributed to the article and approved the submitted version.

## Funding

This work was supported by National Natural Science Foundation of China (82003747), Natural Science Foundation of Tianjin (19JCQNJC12600 and 20JCQNJC00260), and Research project of the Tianjin Education Commission (2019KJ044).

## Conflict of Interest

The authors declare that the research was conducted in the absence of any commercial or financial relationships that could be construed as a potential conflict of interest.

## Publisher's Note

All claims expressed in this article are solely those of the authors and do not necessarily represent those of their affiliated organizations, or those of the publisher, the editors and the reviewers. Any product that may be evaluated in this article, or claim that may be made by its manufacturer, is not guaranteed or endorsed by the publisher.
